# The Support Needs Approach for Patients (SNAP): content validity and response processes from the perspective of patients and nurses in Swedish specialised palliative home care

**DOI:** 10.1186/s12904-025-01715-4

**Published:** 2025-03-18

**Authors:** Carina Lundh Hagelin, Maja Holm, Lena Axelsson, Marjukka Rosén, Terés Norell, Zilmara Suárez Godoy, Morag Farquhar, Gail Ewing, A Carole Gardener, Kristofer Årestedt, Anette Alvariza

**Affiliations:** 1 Department of Neurobiology, Care Sciences and Society, Division of Nursing, Karolinska Institutet, Stockholm, Sweden; 2 Department of Health Care Sciences, Marie Cederschiöld University, Stockholm, Sweden; 3 Department of Nursing Science, Sophiahemmet University, Stockholm, Sweden; 4 Region Stockholm, Advanced home care, Stockholm South, Nacka, Sweden; 5 Region Uppsala, Anaesthetics, surgery and intensive care at Uppsala University Hospital, Uppsala, Sweden; 6 Region Uppsala, Hospital-based home care unit, Enköping hospital, Enköping, Sweden; 7 School of Health Sciences, University of East Anglia, Norwich Research Park, Norwich, UK; 8 Centre for Family Research, University of Cambridge, Cambridge, UK; 9 Faculty of Health and Life Sciences, Linnaeus University, Kalmar, Sweden; 10 Department of Research, Region Kalmar County, Kalmar, Sweden; 11 Department of Research and Development/Palliative Care, Stockholms Sjukhem, Stockholm, Sweden

**Keywords:** Support Needs Approach for Patients, Palliative care, Person-centred care, Translating, Response processes, Content validity

## Abstract

**Background:**

The Support Needs Approach for Patients (SNAP) enables patients to reflect on, identify and prioritise their own support needs from a holistic perspective and enable tailored support. Therefore, the aim of this study was to examine the content validity and response processes for the Swedish version of the SNAP Tool among patients with life-threatening illness and palliative care needs, and registered nurses (RN) in specialized palliative home care services.

**Methods:**

This was a two-stage validation study: (I) translation of the original English version of the SNAP Tool into Swedish, and (II) examination of content validity for patients and RNs in specialized palliative home care, and response processes among the patients. Cognitive interviews were conducted with patients (*n*=11) and focus groups with RNs (*n*=10). Data were, in stage II, analysed for relevance, clarity, and sensitivity.

**Results:**

The translation process identified a few differences in wordings that were thoroughly debated to retain the meaning of the questions. Both patients and RNs considered the Swedish version of the SNAP Tool relevant to the palliative care context and its questions clear and easy to understand. Patients believed that their responses on the tool could be helpful in providing a clear structure for conversations and present a picture of their individual support needs. There were just a few considerations about sensitivity of questions from the patients’ perspectives and the RNs felt that some of the questions may need to be handled with care.

**Conclusions:**

This study demonstrates that the Swedish version of the SNAP Tool has good coverage of Swedish patients’ support needs, and that the questions are perceived as intended. This indicates that the SNAP Tool effectively captures a wide range of support needs and aligns with its intended purpose. The tool is appropriate for specialized palliative home care and allows the SNAP intervention to be made available to this group of patients.

## Background

Living with a progressive and chronic disease is demanding in many ways [1]: patients often face physical, psychological, social, and existential challenges [1–3]. Different types of supportive interventions are needed to enable patients to have the best possible well-being and quality-of-life through to the end of life [2]. It is important that the patient's own experiences, preferences, individual needs, and resources are considered in enabling them to live with dignity [4]. Difficulties in identifying patients' needs have been demonstrated, for example, in the care of patients with chronic obstructive pulmonary disease (COPD) [5] or advanced cancer [6]. Gardener et al found that some patients with COPD under report their need for help despite expressing a desire for more contact with a doctor, indicating some hesitation or difficulty in discussing their concerns. These findings are supported by the work of Chatwin et al (2014) and Chew Graham et al (2013) who similarly exposed difficulties for patients with COPD in reporting their needs and a lack of person-centred consultations [7, 8]. The fact that patients do not fully report their needs may affect how clinicians interpret their needs and thus actions may be missed or misdirected [7, 8]. Physicians who encountered patients with advanced cancer [6] felt that patient-reported outcome measures (PROM) could be useful to facilitate communication about patients' personal values, wishes and needs and improve palliative care in hospital cancer care, but they lacked a systematic approach to discuss these topics.

There has been a considerable effort to develop assessment tools to identify patients’ subjective symptoms and problems to be able to initiate appropriate responses [9, 10]. Using PROMs are suggested as one way to increase patient-centeredness of treatment and improve the quality of palliative care [11]. The use has however, been found to lead to only moderate improvements in clinician-patient communication, diagnosis-, and disease management, and slightly improve quality of life [12]. Patient Reported Experience Measures (PREMs), on the other hand, are developed and used to capture patients’ perspectives about their care and used for development of care for patients on an overarching level, but there is a lack of substantial evidence of impact and evaluations of how these affect individual patient experience [13]. Further, few tools have focused on patients’ own identified support needs. Identifying and addressing support needs is crucial for ensuring safety and security for patients with palliative care needs and their family carers. While home is often seen as the safest place, insecurity can arise due to worsening symptoms, reduced daily living activities, inadequate care, need for domestic services, or insufficient capacity of family carers [14].

The Support Needs Approach for Patients (SNAP) [15, 16] was originally developed to enable patients with COPD to reflect, identify, prioritise and address their needs of support from a holistic perspective. The SNAP is an intervention consisting of two parts: (1) the SNAP Tool and (2) a five-stage person-centred process of assessment, support and review. The SNAP Tool comprises 15 evidence-based questions relating to patients’ unmet support needs (Table 1 shows the domains of support need covered by the questions). The questions are presented in a grid format with three response categories (No/A little more/Quite a bit more) to facilitate the expression of needs by the patient. Within the 5-stage person-centred process, Stage 1 introduces SNAP, in Stage 2 the patient identifies their needs using the SNAP Tool, Stage 3 involves a conversation with a health-care professional about the needs the patients have identified and prioritised. Thereafter a joint action plan is created (Stage 4) and finally a reassessment of needs is conducted (Stage 5) [15, 16].

**Table 1:** The SNAP Tool support domains

**Do you need more support with**
…understanding your illness?
…managing your symptoms (including medication and oxygen)?
…dealing with your feelings and worries?
…looking after any other physical health problem you may have?
…having a healthier lifestyle (e.g., keeping active or eating well)?
…getting out and about?
…overcoming boredom or loneliness?
…financial, legal, work or housing issues?
…practical help in the home or garden?
…your personal care (e.g., dressing, washing)?
…aids or equipment to help you?
…family relationships (including talking to your relatives about your illness)?
…knowing what to expect in the future?
…accessing or using services?
…anything else?
Does a family member or friend who helps you need more support?

Presentation inspired by Gardener, Ewing, Mendonca, & Farquhar (2019) [16]

Given the generic nature of the questions on the SNAP Tool, SNAP is being used with patients with a range of progressive life-limiting conditions. Importantly, the SNAP Tool is not a measure, form, or scale, but a communication tool to open up for a conversation with patients about their specific support needs. This means cannot, and should not, be classically psychometrically tested [16]. However, exploration of the relevance of its content and response processes are required in order to evaluate translated versions of the SNAP Tool, just as in the development of the original English version [16]. Content validity refers to the relationship between the content of the questions and the construct they are intended to cover, while response processes refer to processes of responses to test items [17]. Therefore, the aim of this study was to examine the content validity and response processes for the Swedish version of the SNAP Tool among patients with life-threatening illness and palliative care needs, and registered nurses (RN) in specialized palliative home care services.

## Material and Methods

To ensure the usability of the SNAP tool, we conducted a systematic process of translation, cultural adaptation and preliminary validation. The thorough work was necessary to preserve the intended meaning of each question and maintain semantic, conceptual and idiomatic equivalence in Swedish [18]. A two-stage validation study was performed, including: (I) translation of the original English version of the SNAP Tool into Swedish, and (II) content validity of the translated tool was then examined for patients and registered nurses (RNs) in specialized palliative home care, alongside the response processes of the patients. Cognitive interviews were performed with patients and focus groups (FGs) with RNs.

### Stage I – Translation process

Following issue of a translation licence (https://thesnap.org.uk/use-snap/licensing/), and the recommendation of the developers of SNAP Tool, the tool was translated from English to Swedish following the procedure described by the European Organization for Research and Treatment of Cancer [19], see Table 2, apart from using a professional forward translator.

**Table 2:** The translation procedure

⦁ forward translation to Swedish by two independent native Swedish persons, fluent in English
⦁ a preliminary Swedish version of the tool compiled by two of the authors (CLH, AA)
⦁ independent review by the other three members of the Swedish research group
⦁ meaning of each original question and the meaning in the Swedish translation extensively discussed
⦁ changes made to form an agreed version
⦁ agreed version back translated into English by one professional translator and a native English author fluent in Swedish
⦁ the back-translated version was compared with the English original version by two authors (CLH, AA).

### Expert group

The forward translation process, from English to Swedish, involved seven RNs with Swedish as their mother tongue. Two RNs, native Swedish, performed the forward translation separately, these were then reconciled and discussed in the expert group of five of the authors who possessed extensive clinical and research experience from Swedish health care and validation processes, and fluent in English. When there were one reconciled and agreed Swedish version, a back-translation was made by a licenced translator. Some minor discrepancies were further discussed by two of the authors and the translator. The translation process benefited from the research team's experience translating the Carer Support Needs Assessment Tool Intervention (CSNAT: https://csnat.org/) into Swedish [20], a predecessor to SNAP that was developed for family carers. One of the two original developers of CSNAT-I was also one of the developers of SNAP (Dr G. Ewing) and the other (Prof. G. Grande) speaks and understands Swedish: both participated in, and advised on, the translation of the SNAP Tool.

#### Discrepancies and finalising the translation

A few differences in wordings were identified, for example, “Do you need more support with getting out and about?” (question 10) has no obvious translation into Swedish. Differences in wording were discussed within the expert group, with the professional translator, SNAP developers, and CSNAT-I developers, enabling further refinement of the SNAP Tool’s Swedish translation. For example, in this case, the solution formulated was “Do you need more support getting out of the home?”, with an explanation in parenthesis “(e.g. for activities or to meet people)”. Finally, a preliminary Swedish version was compiled by the expert group and reviewed and approved by the SNAP developers. The SNAP Tool also includes some brief instructions on how to complete the tool. These instructions largely follow the instructions on the CSNAT that has been in use in its translated form in Sweden since 2020, therefore translation of these instructions did not require the same rigorous translation process but were instead forward translated by two of the authors (CLH, AA) and then reviewed by three other authors (MH, LA, KÅ) and the SNAP Team.

### Stage II: Examination of content validity and response processes – patients’ perspective

Content validity and response processes from the patients’ perspective were examined through cognitive interviews [21]. In the cognitive interviews, patients were asked to reflect on the relevance, clarity and sensitivity of each of the tool’s fifteen questions, one at the time. For response processes patients were asked to reason aloud when answering each question. A probe technique was used to elicit participants' understanding and cognitive processes for each question. The data collected consisted of the researchers' notes for each question and suggestions for changes to solve any issues identified [21].

#### Setting

One of the largest specialised palliative home care services in Sweden, which provides multi-professional care for patients aged ≥18years, with life-threatening illness and palliative care needs gave consent to participate.

#### Recruitment of patients

With permission from the head of the service, one of the authors (MR), who was also a RN at the service, identified eligible patients from a purposive recruitment strategy to provide variety in terms of age, gender, marital status and diagnosis. To achieve variation in diagnoses representing palliative care, patients’ medical records were initially screened to identify patients with chronic heart failure, COPD, chronic renal failure, and incurable cancer. At the time of the study, about 80 patients with these diagnoses were enrolled for care and, from these, 15 patients were purposively selected using the inclusion criteria, i.e. being a patient aged ≥18years, with life-threatening illness and palliative care needs. Cognitive impairment and inability to understand and speak the Swedish language were criteria for exclusion. Eligible patients were given oral and written information by the RN (author MR) and thereafter contacted within 7-10 days to ask for their interest in participating. Four patients declined participation due to tiredness and lack of strength. Eleven patients chose to participate, and a time and place was agreed with each patient for individual interviews. The SNAP Tool was sent to patients a week ahead of their interview.

#### Data collection with patients

Patients who agreed to take part were again given information about the study prior to providing written consent.

Background data were collected about the patient’s age, gender, level of education and diagnosis. The 11 participating patients had a variety of life-threatening illnesses and palliative care needs and were cared for in their own homes (Table 3). All but one patient had a cancer diagnosis; five patients had an additional diagnosis. The one patient who did not have cancer had COPD (Table 3). A topic guide for examining content validity had been developed covering the relevance, clarity, and sensitivity of the SNAP Tool questions, as well as its overall layout and response options. This topic guide had been used in an earlier validation study [22] and was pilot tested with one patient which did not lead to any changes; this pilot interview was therefore included in the study data. The cognitive interviews followed Willis’ [21] guidelines: a combination of 'think-aloud' and 'probing questions' techniques were used, as described above, to explore patients’ response processes. All interviews were conducted in patients’ homes during October and November 2021 and audio recorded with permission. Interviews lasted between 13 and 43 minutes.

**Table 3:** Patients’ characteristics (*n*=11)

Age (years), median (min-max)	65 (46–83)
Gender, n (%)	
female	7 (64)
male	4 (36)
Country of birth, n (%)	
Sweden	7 (64)
Nordic countries (other than Sweden)	2 (18)
Europe (other than Sweden and the other Nordic countries)	1 (9)
Other parts of the world	1 (9)
Education, n (%)	
Primary school or equivalent	
Secondary school or equivalent	4 (36)
University	7 (63)
Diagnos, n (%)*	
Cancer	10 (91)
COPD	3 (27)
Heart failure	2 (18)
Renal failure	1 (9)

^*^ All but one patient had any form of cancer. COPD = Chronic Obstructive Pulmonary Disease

The interviewer read each question on the SNAP Tool (one at a time) and then asked the respondent to think aloud spontaneously while answering the SNAP Tool question. Following this they were asked to comment on the question’s relevance, clarity, and sensitivity. Probing questions used were, for example, "What are you thinking when you answer the question?" or "How did you arrive at that answer?" and "What does that word mean to you?". When all questions on the SNAP Tool had been answered, the interviews concluded with questions about the design of the response options, time taken to complete the tool, and the overall layout of the tool.

### Stage II: Examination of content validity – the RNs perspective

Professionals’ perspectives on content validity were examined in focus group discussions [23] with RNs.

#### Setting

The same specialised palliative home care services in Sweden as described above gave consent to invite RNs to focus group discussion. The service provides 24-hour care, with varying numbers of home visits depending on each patient’s care needs. RN is the largest profession in this service.

#### Recruitment of RNs

A convenience sample of RNs was used, but variation was sought in terms of gender, education (general or specialist RN education), and work experience in specialised palliative home care. The responsible RN at the unit (author MR), and the head of the service, proposed two dates and times for the FG discussions. Author MR provided oral and written information about the study and written consent was collected by two authors (ZSG, TN) at the time of the FGs. The SNAP Tool was sent to the RNs two days before the planned FG including instructions on how to use the SNAP Tool in practice (i.e., how to deliver SNAP – the intervention that the SNAP Tool underpins).

#### Data collection with registered nurses

The same topic guide for examining content validity among patients was used for RNs. This was pilot tested with three RNs from another palliative care service, who had worked in palliative care for 5-15 years. The pilot interviews did not lead to any changes but provided an opportunity for the interviewers to become familiar with the topic guide and test open and probing questions to invite interaction and discussions. These data were not included in the study data.

Before the FG discussion started, participants were again informed about the aim of the study, gave written consent, and completed a background information questionnaire which included: age, number of years in the profession, number of years working in specialised palliative care, and whether they had a degree as a general RN or specialist RN (Table 4). FG discussions were facilitated and co-facilitated by two of the authors (ZSG and TN respectively). At the start of the FG, participants introduced themselves by name, after which the facilitator started the conversation using the topic guide, asking about each of the SNAP Tool’s questions (again, read aloud one at a time) in terms of relevance, clarity, and sensitivity. The group then discussed the tool’s instructions, layout, and response options. Two focus groups were conducted with the RNs (*n*=10 in total) between October and November 2021 in a private room at the participants' workplace. The discussions lasted about one hour each.

**Table 4:** RNs characteristics (*n*=10)

	Median (min-max)	Numbers (%)
Age (years), median (min-max)	56 (25 – 66)	
Number of years in the profession (RN)	22 (3 - 45)	
Number of years in specialised palliative care	8 (<1* - 26)	
Undergraduate^a^ RN		6 (60)
Post graduate RN (Specialist exam)		4 (40)

^*^ = worked 10 months in specialised palliative care. ^a^ RNs educated to a general degree level.

### Data analysis

All audio-recorded data were transcribed verbatim. Transcripts were reviewed and checked for accuracy by the author who conducted the interviews (MR, ZSG, TN) and two others from the research group (CLH, MH) by listening to the recordings and reviewing them against the transcripts and interviewer's notes (notes of observations of reactions and behaviour made during all interviews). At this stage, the data was anonymised, and each interview was given a number.

The analysis undertaken was based on Willis’ guidelines [21]. Authors (CLH, AA) read all interview texts and summarised responses to each of the SNAP Tool’s questions to identify content related to relevance, clarity, and sensitivity for patients and professionals, and patients’ response processes. The analysed content was discussed among all authors and coded according to the predetermined categories. Quotes are used to illustrate the analysed content and are reproduced with reference to participants 1, 2, etc., to maintain confidentiality among participants [24].

### Ethical considerations

In palliative care, patients are considered vulnerable, but research shows that they appreciate participating in studies and benefit from their involvement [25]. Clinical RNs may feel judged when discussing their work but can also benefit from such discussions. Participants received written and oral information about the study, including their right to withdraw without affecting patient care. Procedures were put in place to support participants with the interview process. All those who conducted interviews with patients and RNs, were RNs themselves with extensive experience in palliative care. In addition, if any participants became distressed, referral on to further support was available if needed. Data analysis followed confidentiality principles, and the study was approved by the Swedish Ethical Review Authority (no. 2021-0384).

## Results

The results are presented in terms of the SNAP Tool’s relevance, clarity and sensitivity as reflected by both patients and RNs.

### Relevance – the patients´ perspective

Patients found the SNAP Tool’s questions highly relevant to palliative home care but also for healthcare in general. Patients thought the SNAP Tool could be useful to both healthcare professionals and to themselves. They believed that their responses on the tool could be helpful in providing a clear structure for conversations and present a picture of individual patient’s support needs. One patient described having had an earlier conversation with a clinical staff on admission to home care in which none of the question areas on the SNAP Tool had been raised. This same patient suggested that the tool might not be appropriate at the first meeting, but that it would be a good for enabling later discussions. Participants expressed that the tool could be used regularly, with quarterly completion being suggested as monthly might be too often, generating too much work for the clinical staff.

Patients felt that the SNAP Tool’s questions were important to be asked, but also useful for them to reflect on by themselves. They felt the SNAP Tool could help them identify and articulate support needs that otherwise would be difficult to think of and express on their own. At the same time the range of response options allowed patients to take control e.g., of what support needs they wanted to identify at that time. This was described through expressions such as: ‘*It's difficult to answer, I've kind of chosen myself not to know so much. I don't want to know the whole truth yet’ (pt.no 1)*.

Completing the SNAP Tool gave patients the opportunity to think about their situation from different perspectives: it facilitated expression of symptoms or experiences that might change over time. Patients found it positive that not only physical issues but also psychological well-being and social consequences of being unwell were included, acknowledging that illness is about more than just medicine and physical well-being. The questions also awoke reflections about how their illness suddenly turned their whole life upside down with a need to reflect upon one's own situation. The patients did not expect the clinical staff to solve their situations, but the SNAP Tool provided a reminder to patients to be proactive and to manage things themselves. It raised questions such as *‘What can I do*? *Where can I turn to*? *How should I act*?*’ (pt.no 7).*

Patients who did not report a need of support at the moment suggested that the SNAP Tool still provided an opportunity to reflect on how they had already resolved some areas themselves. This included practical help through employing a cleaner or by dividing up housework tasks in order to cope – *‘But yes, I try to plan it in. Today, if I'm going to do a bit of shopping, tomorrow I'll cook (laughs) not every day, I can't be bothered. So, it's hard to stand, I think. But I divide it up, the days and do small things, that's fine’ (pt. no 3).*

Some reflected on the question ‘Do you need more support with overcoming boredom or loneliness?’ (question 7), identifying how experiences of loneliness linked to particular occasions such as when the family were away for a while. One patient reasoned: ‘*But, no, but, yes, you can of course... can be sad at home and lonely too. But you must find your own activities’ (pt.no 2)*.

Patients indicated that there was a need to talk to clinical staff, to not burden relatives too much, and found the SNAP Tool could facilitate this. When patients reviewed the question ‘Does a family member or friend who helps you need more support?’ (the final question on the SNAP Tool, Box 1) it emerged that some did not really know whether their family carers needed support.

### Relevance – the RNs´ perspective

RNs considered that the SNAP Tool could be relevant to enable structured conversations about patients’ self-identified needs, especially concerning needs related to practical support. However, the nurses questioned the relevance of asking patients to respond to questions related to support needs that, they felt, would not be possible for them as RNs, or other health care services, to meet. One example of this was the question focused on financial and housing issues (question 8). Further, the RNs were concerned that patients might be too frail and hence the SNAP Tool would need to be discussed in more than one meeting, which would take more time.

The questions could open up for conversations regarding sensitive areas, such as death, that otherwise might be difficult to initiate. RNs therefore welcomed implementation of the SNAP intervention in specialized palliative care as early as possible in the illness trajectory. The questions in the SNAP Tool were considered comprehensive with the potential to illuminate important aspects of the patients’ lives. This was suggested as especially important with younger patients as they have often not thought about practical and legal issues.

### Clarity - the patients´ perspective

Patients perceived the SNAP Tool as being useful as it was clear, easy to understand and respond to, and sufficiently comprehensive. The fact that the questions were all on one page was appreciated, along with the size of the text and the three response options. There were, however, some concerns about the meaning of the questions about ‘understanding your illness (question 1) and ‘knowing what to expect in the future’ (question 13) which, for these patients, were felt to be somewhat related to each other *– ‘I imagine here, question 1 and 13, they are connected a little bit, I think. "To understand your illness" and "To know more about the future", are a bit close to each other. It can be a bit ... clarified a bit’ (pt.no 8)*. Further, regarding the question about ‘accessing or using services’ (question 14), some patients asked for suggestions to clarify what was meant by “services” whereas others reasoned that they got the help they needed from the hospital or the home care team. Patients highlighted the importance of straightforward and simple language in a tool such as the SNAP Tool in order to facilitate understanding for all.

### Clarity – the RNs´ perspective

RNs also found the SNAP Tool clear overall and easy to understand. However, they were concerned that the question about ‘boredom or loneliness’ (question 7) could be interpreted as including general experience of boredom and loneliness, unrelated to health. They suggested that the word “possible” could be placed before the question. The RNs further suggested that the clarity of the SNAP Tool could be increased by changing the pronouns from *yours* to *my,* i.e., changing the words ‘*your’* to ‘*my’* to make it even more person-centred and explorative. The RNs also considered the term *‘your illness’ (in ‘Do you need more support with understanding your illness?’ Question 1)* to be a medical term, but ´m*y illness situation´* would better encompass potential multimorbidity in the patient.

The RNs considered the three response options on the SNAP Tool appropriate, with a high coverage, and easily distinguishable. Having just three response options was considered clear and sufficient given that patients could be low on energy. The response option ‘*A little bit more’* was considered suitable for patients that might be cautious about expressing their support needs in conversations. The response option ‘*No’* was considered helpful as the patient could clearly state that they had no current need for (or wish to currently discuss) support in relation to that question.

### Sensitivity – the patients´ perspective

Patients did not find any of the questions too sensitive to consider or talk about and thought they were appropriately worded and not upsetting. They involved topics that they had already thought about. Patients also pointed out that the response categories provided the opportunity for them to decide what areas of support need should be discussed by answering ‘No’ to questions they did not want to talk about at that point in time, giving them the control noted above.

Patients reflected that when severely ill, there may be feelings of anxiety involved, and some people can be particularly sensitive. It was therefore suggested that, for some, it might be sensitive to complete and reflect upon the questions alone, depending on how they felt about themselves or their situation. Some specific questions were highlighted as being potentially sensitive in relation to individual perspectives or circumstances, e.g., ‘Do you need more support with having a healthier lifestyle (e.g., being active or eating well)’ (question 5) was suggested to possibly lead to feelings of being judged in cases where an illness might have been influenced by smoking. However, the patient who raised this did not feel the question itself was wrong to ask but, based on an individual person's situation, it could be sensitive and further reasoned about the question as follows: ‘*Since there is not much that can be done about the disease itself, I must try to do something about everything else that can be done reasonably well. And I see it as...well, now there's a deadline on this, that... and I can eat as much Daim [Swedish chocolate] as I want right now (laughs). It's like, it's a parenthesis in life. So, it... yeah. But... I see, what I can do is to think positive, move, rest, and eat well, and then not stress too much’ (pt. no 5).* Patients felt that the question ‘Do you need more support with financial, legal, work or housing issues?’ (question 8) might be experienced as sensitive for those needing support with these issues, in particular financial needs. Also, the question ‘*Do you need more support with family relationships (including talking to your relatives about your illness)?’ (question 12)* was considered as potentially sensitive by some patients, depending on the relationship with relatives.

### Sensitivity – the RNs´ perspective

RNs believed that some of the questions in the SNAP Tool might need to be handled with care as they might be too sensitive and could trigger anxiety or discomfort for patients, e.g., asking about their expectations for the future. They also thought that when working with patients with severe illness and palliative care needs, some of the words used in the SNAP Tool questions could be considered as insensitive for example *health problems, medication and oxygen, aids, and equipment, keeping active and eating well.* They felt the language could be modified to take the palliative care context into consideration as asking about eating well could be experienced as demanding or accusatory by the patients: however, none of the patients raised this concern. The RNs also believed that some questions could be overwhelming for some of their most frail patients, as they might consider it demanding to find solutions to their needs and problems – something that could take a lot of energy.

## Discussion

This is the first study where the SNAP Tool has been translated into another language and evaluated in a specialised palliative home care context and in a broader group of patients, i.e. with different diagnoses than the original English version. The results show that both patients and RNs considered the Swedish translation of the SNAP Tool as relevant to the palliative care context and the included questions were clear and easy to understand. There were just a few considerations about the sensitivity of some of the questions from the patient’s perspective and the RNs felt that some of the questions might need to be handled with care which are discussed further below.

This is, to our knowledge, the first tool in Swedish healthcare that aims to enable patients to reflect, identify and prioritise their own support needs. SNAP (underpinned by the translated SNAP Tool) could give patients the same opportunities that have previously been successfully introduced in Sweden for family carers of patients in palliative home care by the translation of CSNAT-I [26–29]. The SNAP Tool (and the intervention it underpins) represents a significant addition to existing assessment tools [9, 10, 30, 31] as it focuses on patients’ own identification of their support needs. As such it is more person-centred as it allows for holistic assessment of support needs identified by the patients themselves and, through the SNAP intervention, their identification of possible ways to meet these needs.

The present study demonstrates that the SNAP Tool, with its comprehensive intervention, is relevant for patients with various life-threatening illness as well as for RNs in palliative home care. The results are also in line with a validation study of the original English version [16]. The results of the present study also confirm the broader area of applicability of the SNAP Tool and the SNAP intervention among patients in palliative care beyond the original validation for patients with COPD [15, 16].

Interestingly, some discrepancies between patients and RNs views on the tool’s relevance, sensitivity, and clarity were identified. RNs were more cautious, feeling, for example, that the question about practical support was particularly relevant whereas the patients felt that although this question was relevant, the questions about psychological well-being and social consequences were more important and highly relevant as they considered illness as more than a medical and physical condition, reflecting a more holistic perspective. RNs questioned the relevance of including questions about issues they thought that health care professionals could not solve, e.g., if a patient experienced boredom or loneliness due to other aspects that health related. Patients, on the other hand, did not expect RNs to solve these problems but instead valued that the SNAP Tool triggered them to think about and be proactive in these areas, and gave them the opportunity to also identify and reflect on areas they had already solved by themselves. This highlights the importance of SNAP training for clinicians (RNs in this study), so that they fully understand the person-centred SNAP intervention, that the intention is not that RNs are responsible to solve all problems but that responses to support needs can include, for example, signposting or referral on to services that can address the needs. SNAP training is free to access on the SNAP website: https://thesnap.org.uk/use-snap/training/. One important aspect of delivering SNAP (the whole intervention) is that it enables an approach in which the RNs step back from their ‘expert’ or ‘leading’ role and instead open up for opportunities to talk about what is a prioritised concern for the patient. This has also been the experience of using the CSNAT-I with family carers in Sweden [26, 32]. This sits within theoretical frameworks outlining a person-centred approach to care in which the patient is seen as a unique person that is involved, engaged, and allowed to control their care [33].

Both patients and RNs in the present study found the translated SNAP Tool questions and instructions clear and easy to understand. The straightforward and understandable language in the SNAP Tool was highlighted as important. The importance of a plain language without the use of medical terms was also noted by patients in the validation of the original English version [16]. RNs suggested a change in phrasing from ‘your support needs’ to ‘my support needs’, however, patients did not suggest this. We therefore chose to retain ‘your support needs’ to align with the original version of the SNAP Tool, and as the SNAP Tool (like CSNAT), is introduced by clinical staff to patients (or relatives in the case of CSNAT), it is more fitting to introduce a framework for identifying your support needs.

The SNAP Tool was considered to be useful to help guide a conversation, as expressed earlier [5, 15]. This is also in line with the initial validation of the Swedish CSNAT where both family caregivers and RNs believed that the CSNAT-I could facilitate opportunities for family caregivers to express their needs and that it could be used repeatedly during the care [20], which has been demonstrated positively [26–28]. The experience from one patient who highlighted an early conversation with clinical staff at the palliative care service where aspects of the SNAP Tool were not all addressed, shows the potential of the SNAP Tool to provide structure and support equitable care, helping patients identify and articulate support needs that otherwise could be difficult to think of and express on their own. At the same time the range of response options allowed patients to retain control.

RNs expressed concerns that some questions might be intrusive for patients and cause anxiety, e.g. questions about illness and the future needs to be mentioned. However, patients expressed that these questions needed to be asked as they could help them to take control of their own support needs. Patients also expressed that they had thought about most of the question areas but may not have identified where the support could come from. Both patients and carers often have support needs, but clinical staff do not always assess them: by using the SNAP Tool the patient is given the opportunity to raise them [5, 34] to plan for further care. Patients also thought that some questions could be sensitive depending on each person’s individual circumstances, but importantly in these situations, the patients welcomed the opportunity to answer ‘*no’* to a question relating to a support need that they might not be ready to talk about at that point in time, again demonstrating their retention of control. This is a crucial finding enabling the patients feel in control, whilst RNs were more cautious and were concerned not to harm the patients: they felt that some questions could be too sensitive to ask patients in a palliative stage of a life-threatening disease. This relates to ethical principles, e.g., autonomy, beneficence, and non-maleficence and might be a source of conflict for the RNs. Patients have varying experiences and preferences regarding conversations, particularly confidential conversations [35]. Patients seek autonomy in choosing confidants and look for trust and comfort in their interactions with RNs. Trust is crucial for creating a safe space for patients to express themselves openly. A sense of belonging can be fostered through conversations with RNs, providing relief from life's challenges. However, feeling unheard or rejected by a RN can deepen loneliness, causing individuals to withdraw and remain silent [35]. This highlights the difficulty in initiating or refraining from deeper conversations with patients and could be further challenged by the timing of delivering the SNAP Tool and its conversation. This may relate to the patients’ descriptions that some areas might not be appropriate to talk about at the first meeting but could enable later discussions. It is important for patients to engage and establish a partnership with healthcare providers, while also taking control to manage challenges related to illness, treatments, and care. Key elements of a person-centred approach include being seen, heard, believed, and treated respectfully both as a person and a patient [36]. This again highlights the importance of clinical staff completing SNAP training and the need to trust patients’ abilities to make their own decisions, but also for the clinical staff to thoroughly consider whether the completion of the tool and reflection should be done by the patient alone or together with someone – if done with someone, it is important that it is the patient’s own reflections and identified needs are considered.

Regarding words, aspects of meaning are likely to be perceived differently by different patients and the conversation that is one part of the SNAP intervention facilitates interactive communication and can enable a trustful atmosphere preventing misunderstandings and deepening the understanding of patients' support needs. This has been confirmed in studies exploring the CSNAT-I, which uses the same person-centred process as the SNAP intervention, and where conversations were found to be important and appreciated by both family caregivers and RNs [26, 27]. The apparent simplicity of tools such as the SNAP Tool might suggest that it would be easy to use in clinical practice without training, however the importance of completing SNAP training (freely available online at the website presented earlier) before using it cannot be over emphasised. This training, which is mandatory for issue of a licence to use the SNAP Tool in clinical practice, enables effective implementation in practice [15, 37]. However, collaborative working is advised within units where SNAP will be implemented. Training in communicating about existential experiences and sensitive topics is helpful to develop sensitivity in approaching patients and initiating dialogue [35, 38, 39].

### Strengths and limitations

Translating any instrument, tool, or scale into a second language is a rigorous process that requires going beyond the literal wording to a translation that captures the meaning of the original language to achieve equivalence between the two languages [40], as demonstrated by terms where no equal translation could be made but the meaning and content needed to be discussed. A strength in this study is the rigorous translation process that was carefully conducted and transparently described in preparation for the empirical work. During this process we noted that extensive discussions were required to maintain the content of each SNAP Tool question beyond a mere linguistic translation. We followed the defined translation process by Koller [19], with the exception of forward translation, for which we did not use a licenced translator but took advantage of the translation of the CSNAT that had been made earlier. Further, in the back-translation stage where two independent translators are usually recommended, a licensed translator instead completed the back-translation following in-depth discussions within the research group including the original developers of the SNAP Tool. Specific emphasis was put on discussions to retain the evidence-based content of the SNAP Tool, i.e. for *“Do you need more support with getting out and about?”* there was no obvious direct translation, so we took time to discuss the question to maintain its meaning in translation. It was valuable to include a bilingual professional translator to find words with a similar meaning between the two languages to achieve an appropriate cultural adaptation of the SNAP Tool. Another strength is that we gathered the views of both patients and RNs. Four patients declined to participate; however it is important to note that they declined participation in the research study and not for clinical use of SNAP to identify their support needs. Finally, using cognitive interviews enabled us to examine the response processes i.e., how patients with life-threatening illness and palliative care needs, in specialized palliative home care services, think and feel while interpreting and responding to the different questions in the SNAP Tool. This, together with the focus group discussion with RNs, supported establishment of the content validity of the SNAP Tool in a new context [41]. The SNAP Tool is not a measurement instrument and therefore no classical psychometric testing was conducted.

The Swedish version of the SNAP tool can provide clear benefits as a research instrument in studies examining patient experiences in palliative care. However, it is also important to recognize its potential in clinical practice to identify and facilitate person-centred discussions regarding patients' support needs. The study demonstrates that the tool is suitable and acceptable for both patients and nurses, with relevant content and a simple format. The tool can assist healthcare professionals in engaging with patients and enable the identification of unmet needs, leading to more tailored support. The next step is to explore how the tool can be utilized in clinical consultations.

## Conclusion

This study rigorously translated the evidence-based SNAP Tool into Swedish and confirmed that the translated tool has good content validity for Swedish patients requiring specialist palliative care. This allows the SNAP intervention (which the tool underpins) to be used with this group of patients and warrants its evaluation in this context in future studies. The findings emphasize the need for clinicians to complete SNAP training before implementing the SNAP intervention.

## Data Availability

The data analysed during the current study are not publicly available as no datasets were generated, but information is available from the corresponding author on reasonable request. For information about SNAP, SNAP training, SNAP licencing, and other translations see https://thesnap.org.uk/

## Abbreviations

CSNAT: The Carer Support Needs Assessment Tool
CSNAT-I: The Carer Support Needs Assessment Tool Intervention
COPD: Chronic obstructive pulmonary disease
RN: Registered nurses
SNAP: Support Needs Approach for Patients
